# A Method for Straightening Distorted Giga-Cast Large Thin-Walled Components

**DOI:** 10.3390/ma17102241

**Published:** 2024-05-10

**Authors:** Donghwi Park, Joonhee Park, Naksoo Kim

**Affiliations:** Department of Mechanical Engineering, Sogang University, Seoul 04107, Republic of Korea; pdhwi93@sogang.ac.kr (D.P.); yory11@sogang.ac.kr (J.P.)

**Keywords:** distortion straightening, thin-walled structures, die-casting, giga-casting, machine learning, finite element analysis

## Abstract

Giga-casting, a revolutionary approach for manufacturing large, single-piece car body components from aluminium, has emerged as a potential game-changer in the automotive industry. However, these large, thin-walled castings are prone to distortions during solidification and heat treatment processes. Straightening these distortions is crucial to ensure structural integrity, facilitate downstream assembly, and maintain aesthetic qualities. This paper proposes a novel method for straightening giga-cast components using a multi-pin straightening machine. The machine’s versatility stems from its ability to adapt to various geometries through multiple strategically controlled straightening pins. This paper introduces the concept of a “straightening stroke decision algorithm” to achieve precise straightening and overcome the challenges of complex shapes. This algorithm determines the stroke length for each pin, combining a polynomial model representing the global stiffness of the component with a machine learning model that captures the stiffness changes arising from the current geometry. The effectiveness of the proposed approach is evaluated through comprehensive numerical experiments using finite element analyses. The straightening performance is assessed for the straightening algorithm with different machine learning models (deep neural network and XGBoost) and compared to a traditional optimisation method. The proposed surrogate models decided the straightening strokes so that the maximum remaining distortion became 0.02% of the largest dimension of each target geometry. The results of the numerical experiment showed that the proposed straightening method is suitable for straightening distortion in large thin-walled components.

## 1. Introduction

The automotive industry witnessed a significant shift with the emergence of giga-casting, a revolutionary approach pioneered by Tesla [1]. This technology utilises colossal, high-pressure die-casting (HPDC) machines, aptly named Giga Presses, to manufacture large, single-piece chassis components from aluminium [2,3]. Prior to giga-casting, car bodies were traditionally constructed by welding together numerous smaller parts. By eliminating the need for extensive welding processes, giga-casting offers the possibility of significantly reduced production times and costs [4]. Additionally, using single-piece components can enhance structural integrity and improve vehicle performance metrics like weight and, potentially, fuel efficiency [5]. As the electric vehicle market continues its rapid expansion, giga-casting’s ability to optimise production for lighter, more efficient car bodies positions it as a potentially disruptive force [6].

Despite the numerous advantages of giga-casting, the process has its challenges. One significant hurdle lies in the inherent tendency for these large, thin-walled components to warp and distort during solidification or subsequent heat treatment processes [7]. Several factors contribute to this phenomenon. The immense size of the castings creates uneven cooling rates across the part, leading to thermal stresses that can cause warping [8]. Additionally, the high pressures employed during casting and the potential for non-uniform heating during heat treatment can induce residual stresses within the material [9]. These combined effects can result in components that deviate from their intended geometry.

Straightening these distortions is crucial for several reasons. Firstly, deviations from the designed shape can compromise the structural integrity of the component [10]. Uneven load distribution and stress concentrations can arise due to warping, potentially leading to safety hazards. Secondly, distorted components can create challenges during downstream assembly processes. Misaligned components can require additional processing and can even lead to production line disruptions [11]. Finally, deviations from the intended form can negatively impact the aesthetic qualities of the final product.

Traditional straightening methods, which involve mechanical pressing, often rely on custom-designed die blocks that match specific sections of the component. These die blocks are expensive to manufacture and can only be used for a particular component geometry [12]. This dependence on custom tooling renders traditional methods incapable of addressing the general complex geometries encountered in giga-castings. The sheer variety of shapes and sizes within these large components would necessitate an extensive and costly collection of die blocks, making traditional straightening impractical for large-scale production. In addition, most of the straightening process has been traditionally carried out by workers’ manual handwork [13], and a systematic approach for the straightening process has yet to be proposed.

Given the limitations of traditional techniques and the growing need for efficient straightening solutions for versatile geometries, this paper proposes a novel approach utilising a straightening machine with multiple straightening pins. This innovative method offers several advantages over conventional methods. Unlike die blocks, which require a custom design for each specific geometry, a straightening machine with multiple straightening pins can readily adapt to a wide range of complex shapes encountered in giga-castings. By strategically pressing the component with each straightening pin, this method allows the distortion to be removed after straightening. It enables the machine to address distortions in specific areas of the giga-casting without introducing new ones elsewhere, making it a highly versatile solution.

Machine learning has emerged as a powerful tool for tackling complex challenges in various mechanical engineering domains [14]. This study utilised two prominent algorithms, deep neural network (DNN) and XGBoost, to address the intricate problem of giga-casting straightening. With their ability to learn complex relationships from vast datasets [15], deep neural networks are well suited for capturing the non-linear interactions of geometric variations in giga-castings. XGBoost, a robust gradient boosting algorithm, excels in handling high-dimensional data [16] and modelling intricate feature interactions [17], making it suitable for predicting stiffness variations within the component. Compared to traditional methods that rely on simpler models or that lack the ability to adapt to complex geometries, incorporating DNN and XGBoost can enhance the straightening performance, leading to more accurate and efficient correction of distortions in these large, complex components.

Numerous researchers have successfully adopted machine learning algorithms for mechanical engineering problems. Salb et al. [18] proposed a method for enhancing IoT network security by combining CNNs for feature extraction with XGBoost for intrusion detection. They further introduced a modified Reptile Search algorithm for hyperparameter optimisation, leading to a more robust defence against emerging threats in IoT security. Park et al. [19] proposed a method for designing patterns in tubular robots that utilises deep neural networks to extract key features and metaheuristics optimisation to achieve desired mechanical properties, surpassing previous designs in performance and efficiency. Ref. [20] developed a system for the intelligent fault diagnosis of rotary machinery using a convolutional neural network (CNN) with automatic hyperparameter optimisation via Bayesian optimisation, achieving accurate fault detection without manual network configuration. Lin et al. [21] combined finite element simulation to generate data on self-piercing riveted joints and utilised the XGBoost algorithm to analyse it, achieving highly accurate predictions of their cross-tension strength with an impressive error rate of only 7.6%, which offered a significant advancement in predicting joint performance, potentially replacing traditional testing methods. Hashemi et al. [22] utilised machine learning to create surrogate finite element models. The surrogate finite element models could efficiently predict the dynamic response of mechanical systems, significantly reducing the time and resources needed compared to traditional full-scale finite element analysis.

This paper introduces the concept of a “straightening stroke decision algorithm” to achieve precise straightening and overcome the challenges associated with complex geometries. This innovative algorithm plays a crucial role in determining the optimal straightening stroke for each individual pin within a straightening machine. The algorithm utilises the following two key components: The first component is a polynomial model representing the global stiffness of the giga-casting component. This model provides a baseline understanding of the component’s overall stiffness to the deformation. The second component is a machine learning model that captures the stiffness changes arising from the current geometry of the specific component being straightened. This model accounts for the deviations of the stiffness of the current geometry from the global stiffness. By combining these two models, the straightening stroke decision algorithm calculates the optimal stroke length required for each straightening pin. The following sections will delve deeper into the details of the straightening stroke decision algorithm, explaining the underlying models and their role in achieving efficient and accurate straightening of giga-cast components.

This paper presents the results of comprehensive numerical experiments to evaluate the effectiveness of the proposed straightening approach. Finite element analyses were employed to simulate the straightening process for various target component geometries. These target components included a simple box, a centre spine, and a side member, each representing different levels of geometric complexity encountered in giga-castings.

The straightening performance was meticulously assessed by comparing the following four different approaches: The first and second approaches are the straightening algorithms with the deep neural network (DNN) and the XGBoost [23] models. These models utilise machine learning algorithms to capture the intricate stiffness variations due to the geometry deviations. The third approach is the straightening algorithm with only the polynomial model for the global stiffness, which means that the model does not consider the effect of the geometry deviations. The last approach is the naive L-BFGS-B method. It serves as a benchmark representing a traditional optimisation technique commonly used for solving engineering problems. By comparing the straightening performance of these approaches across various component geometries, this paper aims to demonstrate the effectiveness of the proposed straightening stroke decision algorithm.

## 2. Distortion Straightening Method

### 2.1. Straightening Machine Concept and Straightening Process

**Figure 1:**
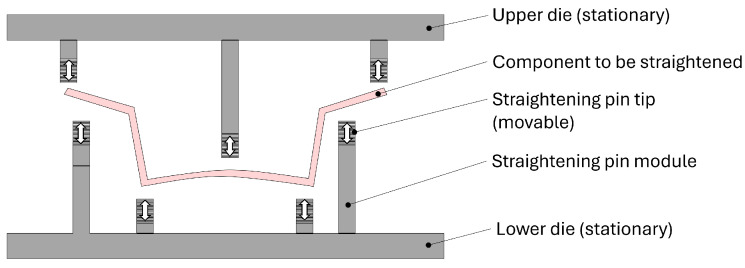
Schematic concept of the straightening machine suggested in this study and its straightening stroke.

The design of the straightening machine suggested in this study is described in Figure 1. The straightening machine has upper and lower dies and numerous straightening pin modules attached to the dies. The lower and upper dies are stationary. The number of straightening pin modules varies depending on the shape of the part to be straightened. The feature of the straightening pin module is that the tip of the straightening pin can move, which makes the machine able to handle various distortions. The component is straightened by pressing it using the strokes of the straightening pins. The movement of the straightening pin can be achieved electrically, pneumatically, hydraulically, or through any other mechanism that can manage the height with enough precision; however, the module system should have appropriate stiffness so that the height is not altered while pressing the part.

The shape of the straightening pin’s tip can be any shape. It can be cylindrical, hemispherical, the local geometry of the part, or any shape suitable for straightening. It is recommended for the straightening pins to be located at the positions where the part has higher stiffness in the press direction. If a pin is located at a position with lower stiffness in the press direction, unwanted local deformation can be generated to the part during the straightening process.

**Figure 2:**
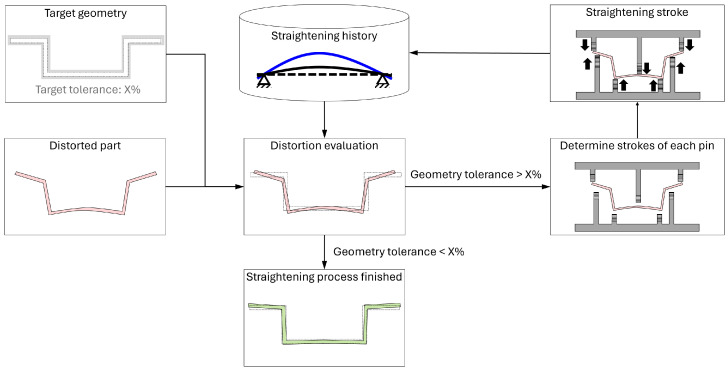
Flow chart of the straightening process.

The procedure of the straightening process is depicted in Figure 2 in the form of a flow chart. The straightening process aims to make the geometry of the distorted part as close as possible to the target geometry so that the dimensional tolerance is satisfied. The first procedure is to evaluate the distortion of the part by comparing the current geometry of the part with the target geometry. If the distortion satisfies the dimensional tolerance, the straightening does not need to be conducted, which means the process is finished. However, if the distortion is out of the dimensional tolerance, the distortion should be straightened. In this scenario, we should determine how much each pin should press the distorted part based on the measured distortion. Once the strokes of each pin are decided, the straightening stroke is conducted on each pin, and the distortion is straightened by the pins pressing the part. When the straightening stroke is finished, the process moves back to the distortion evaluation phase, and the straightening result is saved in the history database to improve the straightening process.

### 2.2. Quantification of Distortion

The distortion of a component can be defined in various ways. In this study, distortion is defined and quantified in the following manner.

**Figure 3:**
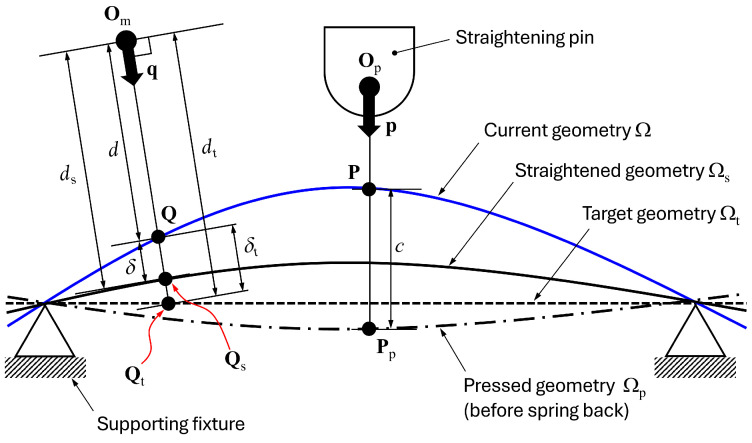
Schematic of a straightening stroke. The current geometry Ω is pressed by a straightening pin in the direction **p**, and the geometry becomes the pressed geometry $\Omega_{p}$. After unloading, $\Omega_{p}$ becomes the straightened geometry $\Omega_{s}$ due to spring-back.

Consider a current geometry Ω and the target geometry $\Omega_{t}$ shown in Figure 3. There is a supporting fixture which prevents the rigid body motion of the geometries. From the measurement origin $O_{m}$, there are intersection points **Q** and $Q_{t}$ with Ω and $\Omega_{t}$ in the direction of measurement **q**. Let the distances from $O_{m}$ to **Q** and $Q_{t}$ be *d* and $d_{t}$, respectively, and define these distances as the distortion measurements. Based on this conceptualisation, in this paper, the distortion is quantified through the difference of the distortion measurements *d* and $d_{t}$ as follows:

(1) $$\delta_{t}=d_{t}-d.$$

$\delta_{t}$ represents how the current geometry should be corrected at point **Q** in the direction of **q**, which means that $\delta_{t}$ can represent how the current geometry is distorted from the target geometry.

The suggested distortion measurement can be easily measured in real situations. A tactile probe can be adopted as an example of a contacting measurement, and laser distance or displacement sensors are examples of non-contacting measurements. In addition, laser scanners could also be an example that rapidly measures the distortion over a wide range.

### 2.3. Straightening Stroke Decision Algorithm

The decision process of the pins’ heights is one of the essential elements in the straightening process since the pins’ heights determine how the part deforms. The straightening stroke decision algorithm proposed in this study decides how much the part should be pressed at the straightening pin locations. The following is a detailed description of the straightening stroke decision algorithm.

Consider a straightening stroke that a straightening pin presses the current geometry Ω from the origin $O_{p}$ with a direction **p** until the geometry becomes the pressed geometry $\Omega_{p}$ as shown in Figure 3. From the initial contact point **P** between the pin and Ω, the pin moves the distance *c* until the contact point $P_{p}$ of $\Omega_{p}$. The pressed geometry $\Omega_{p}$ will be changed to the straightened geometry $\Omega_{s}$ after unloading due to spring-back. The straightening stroke decision algorithm aims to find the distance *c* that makes the straightened geometry $\Omega_{s}$ as close as possible to the target geometry $\Omega_{t}$.

How the current geometry is corrected can be quantified using $d_{s}$, which is the distance between the measurement origin $O_{m}$ and the intersection point $Q_{s}$. δ, the correction amount at the measurement point **Q**, is defined as follows:

(2) $$\delta =d_{s}-d.$$

δ means how the distortion measurement changed at **Q** after a straightening stroke. It can be said that the component is straightened correctly at the measurement point **Q** if the measured δ is close enough to $\delta_{t}$, which is defined in Equation (1). We consider the component straightened when all δ are close enough to their corresponding $\delta_{t}$ since there are multiple straightening pins and measurement points. In other words, the algorithm aims to find the set of *c* values that makes all δ as close as possible to their corresponding $\delta_{t}$.

The straightening stroke decision algorithm utilises a surrogate model that approximates the correction amount δ for the distance *c* based on the current geometry. Say that there are *n* measurement points and *m* straightening pins. In the surrogate model, at the *i*-th measurement point, the correction amount $\delta_{i}$ is modelled as an *N*-th order polynomial based on the assumption that $\delta_{i}$ is a function of the distance *c* and the current geometry. The *N*-th order polynomial for $\delta_{i}$ can be expressed as follows:

(3) $$\delta_{i}={\left(d_{s}\right)}_{i}-d_{i}=\sum_{j=1}^{N}\left(\sum_{j_{1}+\cdots +j_{m}=j}a_{j_{1}\cdots j_{m}}c_{1}^{j_{1}}\cdots c_{m}^{j_{m}}\right),$$

where $c_{k}$ denotes the *k*-th pin movement distance, and *a* denotes a coefficient of the polynomial. The parameter *j* in Equation (3) starts from one since the constant term is neglected in the model. Equation (3) can be expressed as the following equation using vector notation:

(4) δ=KC,

where δ and **C** are the vectorised $\delta_{i}$ and polynomial combinations of $c_{k}$, respectively, and **K** is the matrix representing the polynomial coefficients. **K** is called the stiffness matrix in this study since it represents the “stiffness” of the current geometry. δ has *n* components, while the number of components of **C** varies which depends on the order *N*. The number of components of **C** is calculated as follows:

(5) $$\dim\left(C\right)=\sum_{r=1}^{N}\frac{(m+r-1)!}{(m-1)!r!},$$

which is the sum of the combinations with repetition of *r* objects from *m* objects.

**Figure 4:**
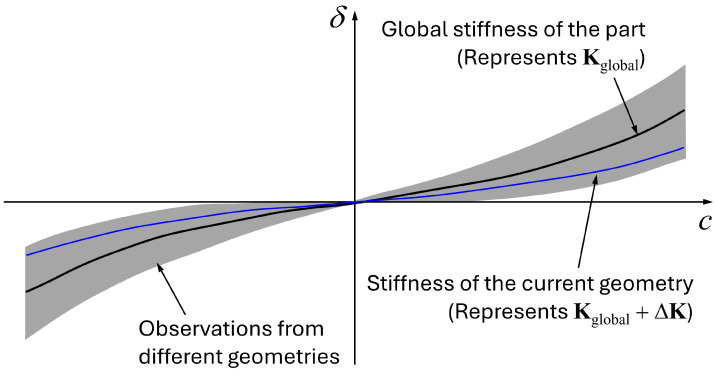
Schematic representation of the global stiffness matrix and the stiffness matrix of the current geometry.

The stiffness matrix **K** strongly depends on the current geometry. It is assumed that the stiffness matrix can be split into two terms, as follows, to consider the effect of the current geometry:

(6) $$K=K_{\mathrm{global}}+\Delta K,$$

where $K_{\mathrm{global}}$ represents the global stiffness of the component, and ΔK represents the change in the stiffness from the global stiffness for the current geometry. Figure 4 shows the schematic representation of the global stiffness matrix $K_{\mathrm{global}}$ and the current geometry’s stiffness matrix $K_{\mathrm{global}}+\Delta K$. The role of $K_{\mathrm{global}}$ is to represent the global stiffness of the part regardless of how the current geometry differs from the target geometry. Assuming that the material properties are constant, how the current geometry looks determines the change in the stiffness matrix, which means that the stiffness matrix of the current geometry can be represented as the sum of these two matrices.

The global stiffness matrix $K_{\mathrm{global}}$ is determined so that the following objective functions $f_{1}$, ⋯, $f_{n}$ are minimised:

(7) $$f_{i}=\frac{1}{M}\sqrt{\sum_{p=1}^{M}{\left[\delta_{i,p}^{\mathrm{measured}}-{\left(K_{\mathrm{global}}C_{p}\right)}_{i}\right]}^{2}},$$

where i=1,⋯,n is the distortion measurement number, p=1,⋯,M is the observation number, and the superscript “measured” denotes the measured value.

ΔK, the change in the stiffness matrix, is modelled as a function of the distortion measurements of the current geometry since the distortion measurements can represent the current geometry. We adopted machine learning algorithms for modelling ΔK since the correlation between the current geometry and ΔK might be highly complex. The objective functions $g_{1}$, ⋯, $g_{n}$ to be minimised for the training of the machine learning models are

(8) $$g_{i}=\frac{1}{M}\sqrt{\sum_{p=1}^{M}{\left[\delta_{i,p}^{\mathrm{measured}}-{\left(\left\{K_{\mathrm{global}}+\Delta K\right\}C_{p}\right)}_{i}\right]}^{2}},$$

where $g_{i}$ is the *i*-th objective function of the *i*-th machine learning model which is for an *i*-th row of ΔK. Note that $K_{\mathrm{global}}$ is fixed during the training of the machine learning algorithm. Two machine learning algorithms were adopted to model ΔK, and a detailed description of these machine learning models is in Section 3.3.

**Figure 5:**
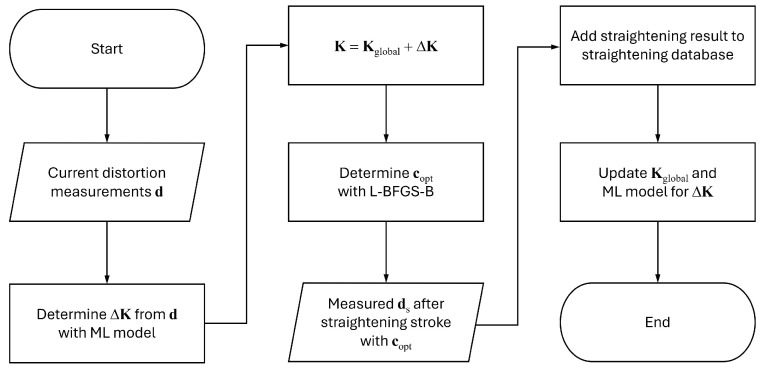
Flow chart of the straightening stroke decision algorithm. ML is an abbreviation of machine learning.

The flow chart of the straightening stroke decision algorithm is depicted in Figure 5. The algorithm starts with input **d** and the vectorised current distortion measurement $d_{i}$. The algorithm determines ΔK from **d** with the machine learning model and calculates the current stiffness matrix **K**. $c_{\mathrm{opt}}$ is the optimum **c**, where **c** is the vectorised pressure distance $c_{k}$, and the subscript “opt” denotes the optimum value. $c_{\mathrm{opt}}$ is determined through an optimisation process with the following objective function $f_{c}$:

(9) $$f_{c}=\max\left(\left|\delta^{\mathrm{pred}}-\delta_{t}\right|\right)=\max\left(\left|KC-\delta_{t}\right|\right),$$

where $\delta_{t}$ is vectorised ${\left(\delta_{t}\right)}_{i}$, and the superscript “pred” denotes the predicted value from the surrogate model. Plenty of optimisation algorithms can be used for the optimisation process of $f_{c}$. In this paper, L-BFGS-B [24] is utilised for the optimisation. The straightening stroke is conducted with $c_{\mathrm{opt}}$, and the distortion measurements of the straightened geometry come into the algorithm. The straightening result is saved to the straightening database. $K_{\mathrm{global}}$ and the machine learning model for ΔK are updated with the updated database, and the algorithm ends. The proposed algorithm can be considered a self-learning algorithm since it can improve its performance by itself.

## 3. Numerical Experiments

### 3.1. Target Components, Straightening Pins, and Distortion Measurement Locations

**Figure 6:**
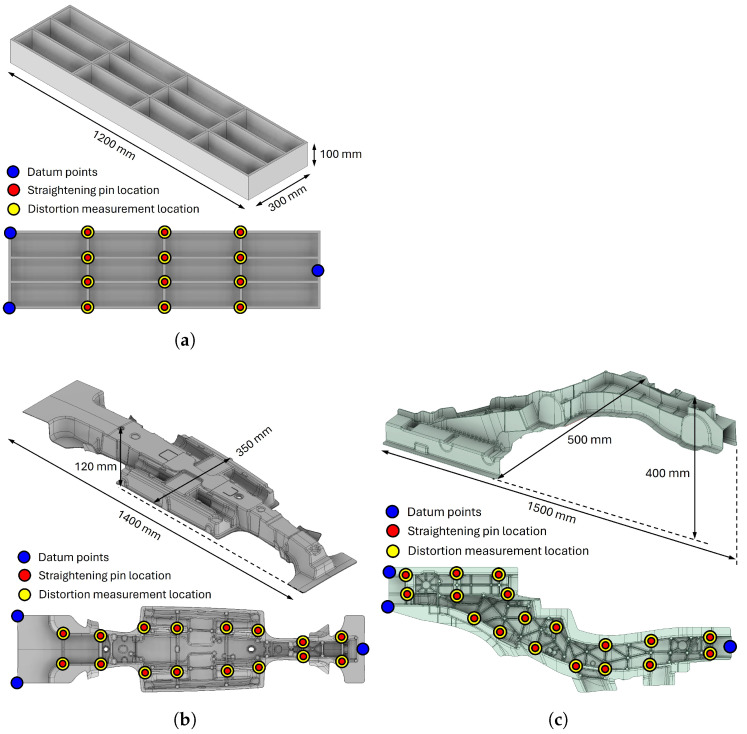
Geometries of the components adopted for the numerical experiments. (**a**) Simple box. (**b**) Centre spine. (**c**) Side member.

Three thin-walled components, shown in Figure 6, were adopted to show the performance of the proposed distortion straightening method. The names of the components are “simple box”, “centre spine”, and “side member” in the sequence of Figure 6a–c. The simple box is a box shape with ribs, which was chosen to represent the very simple geometry of a thin-walled die-cast component. Meanwhile, the centre spine and the side member geometries are from the currently manufactured car frames using the die-cast process.

As the size of the component becomes larger, the possibility becomes higher that the component has a substantial amount of distortion. As a result, the straightening process is likely to be applied to large thin-walled structures; therefore, the die-cast products with the largest dimension above 1200 mm were chosen as the target components. The simple box shape has the largest dimension of 1200 mm and the most straightforward geometry among the target components, so one would expect it to be the easiest part to straighten. The centre spine has the largest dimension of 1400 mm. The centre spine is expected to have intermediate difficulty since the height difference is less than the side member and has symmetric geometry. The side member could be the most challenging component among the target components because it has no symmetric geometry, and the height difference is higher than the two other geometries.

The locations of the straightening pins and the distortion measurements are also depicted in Figure 6. Blue markers indicate the datum points for the distortion measurement. Red and yellow markers indicate the locations of the straightening pins and distortion measurements, respectively. In this study, we only considered cases in which straightening pin locations and distortion measurement locations were identical. On each pin location, two pins were located on the front and back sides so that the components could be pressed from both sides. The distortion measurement direction **q** and the press direction **q** were parallel to the direction of view of the diagrams describing the straightening pin and distortion measurement locations in Figure 6 for all components. The locations were chosen at the point of intersection of the ribs, which had higher stiffness, and the distance between the straightening pins was greater than 80 mm so as to not make them too close to each other. The number of locations was 12 for the simple box, 16 for the centre spine, and 18 for the side member.

### 3.2. Numerical Experiment Procedure

**Figure 7:**
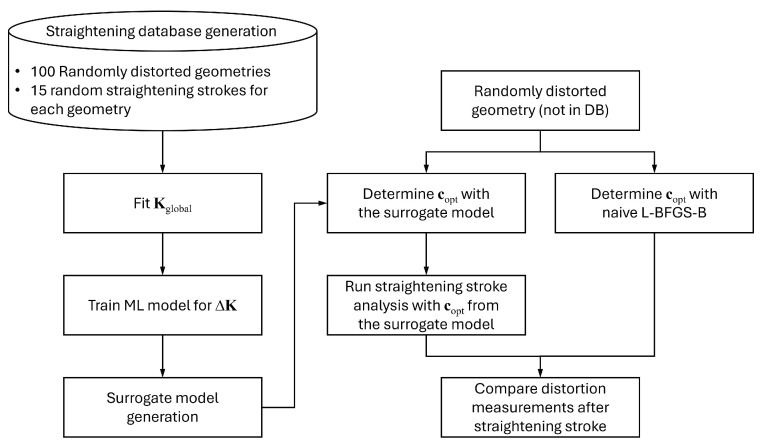
Flow chart of the numerical experiment procedure.

The numerical experiment procedure is depicted in Figure 7 as a flow chart. The first procedure is to generate a straightening database for the surrogate model. The database has 1500 straightening data points, which consist of 15 random straightening strokes of each of 100 randomly distorted geometries. The random distortion of the geometry was generated by pressing the part randomly with the straightening pins. Each randomly distorted geometry’s maximum absolute distortion measurement ranges from 6 mm to 9 mm. Once the database is constructed, $K_{\mathrm{global}}$ is fitted, and the machine learning model for ΔK is trained with the fitted $K_{\mathrm{global}}$. The polynomial order for $K_{\mathrm{global}}$ was chosen as N=3. The number of data points in the database was chosen as 1500 since the minimum required number of data points for 18 straightening pins with a third-order polynomial model is 1329 according to Equation (5).

The performance of the constructed surrogate model was evaluated by comparing the straightening results with naive L-BFGS-B as shown in the right side of the flow chart in Figure 7. The L-BFGS-B algorithm is a quasi-Newton algorithm that aims to minimise a scalar function *f*. For a given $x_{k}$, the position at the *k*-th iteration, the quantities are defined as follows:

(10) $$\begin{array}{cc} s_{k} & =x_{k+1}-x_{k}, \end{array}$$

(11) $$\begin{array}{cc} y_{k} & =g_{k+1}-g_{k}, \end{array}$$

(12) $$\begin{array}{cc} \rho_{k} & =\frac{1}{y_{k}^{T}s_{k}}, \end{array}$$

where $g_{k}=\nabla f\left(x_{k}\right)$ denotes the gradient of *f* at $x_{k}$. The algorithm evaluates the approximate Newton’s direction $d_{k}$ as follows:

(13) $$d_{k}=-H_{k}g_{k},$$

where $H_{k}$ is the inverse of the Hessian matrix. At the *k*-th iteration, the algorithm finds the step length which minimises the function *f* in the direction of $d_{k}$. For the next iteration, the update of the inverse of the Hessian matrix is conducted as follows:

(14) $$H_{k+1}=\left(I-\rho_{k}s_{k}y_{k}^{T}\right)H_{k}\left(I-\rho_{k}y_{k}s_{k}^{T}\right)+\rho_{k}s_{k}s_{k}^{T},$$

where **I** denotes the identity matrix. Naive L-BFGS-B means conducting the L-BFGS-B algorithm to find the optimum pressing distances $c_{\mathrm{opt}}$ by changing the pressing distances in a finite element model, which can be a computationally costly and ineffective method.

Ten randomly distorted geometries, which are not included in the straightening database, were generated and utilised for the performance evaluation of the proposed straightening stroke decision algorithm. The geometries for the performance evaluation also have maximum absolute distortion measurement ranges from 6 mm to 9 mm, which is the same as the database.

**Figure 8:**
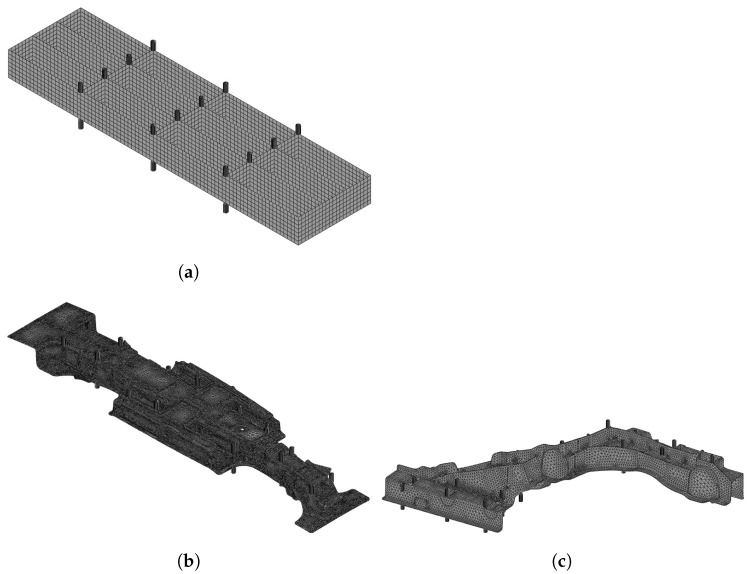
Finite element models of the target components. Each straightening pin location has a pair of straightening pins. (**a**) Simple box. (**b**) Centre spine. (**c**) Side member.

The finite element models were established to conduct the simulations to make the database, conduct the straightening stroke with $c_{\mathrm{opt}}$ calculated by the surrogate model, and conduct the naive L-BFGS-B. The finite element models of the simple box, the centre spine, and the side member are depicted in Figure 8. As shown in the figures and mentioned in Section 3.1, there are pairs of straightening pins at each straightening pin location. The finite element models were developed using the commercial finite element analysis software Abaqus 2020 HF6. The simple box model (Figure 8a) has 4680 nodes and 4655 linear quadrilateral shell with reduced integration (S4R) elements. The centre spine model (Figure 8b) has 235,138 nodes and 767,196 linear tetrahedral (C3D4) elements. The side member model (Figure 8c) has 48,182 nodes and 159,466 C3D4 elements. The straightening pins are set as a rigid body.

### 3.3. Machine Learning Models for ΔK

As mentioned in Section 2.3, ΔK, the change in the stiffness matrix, was modelled using machine learning models. Two machine learning models, a deep neural network (DNN) model and XGBoost [23] model, were adopted to model ΔK. The DNN and XGBoost were chosen since these are known to have satisfactory performance on regression problems [22,25].

**Figure 9:**
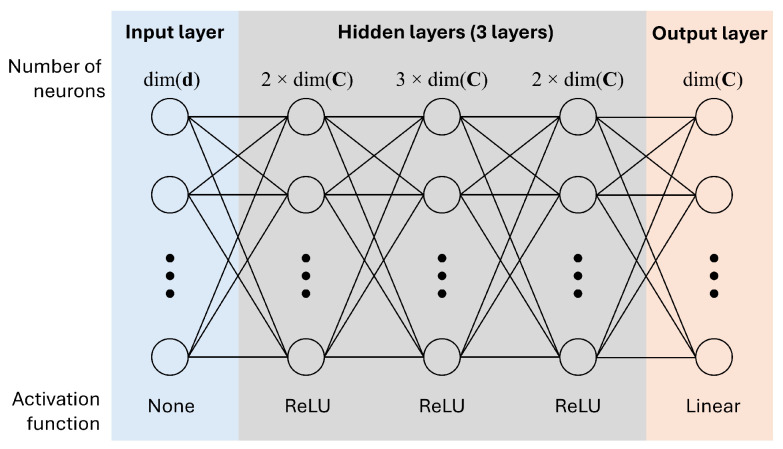
The deep neural network (DNN) architecture for modelling ΔK.

The architecture of the developed DNN model is described in Figure 9. The DNN model was established with Keras [26] on Python 3.9. The input of the DNN model is the current distortion measurements **d**, and the output is a row of ΔK. There are three hidden layers with sizes of 2×dim(C), 3×dim(C), and 2×dim(C). The number of hidden layers was chosen as three based on the work of Asghari et al. [27] since the DNN model with three hidden layers showed fine performance on the regression problem. The rectified linear unit (ReLU) activation function [28] was adopted for the hidden layers. The ReLU function is defined as follows:

(15) f(x)=max(0,x).

The training was conducted using Adam optimiser [29] with a learning rate of 0.0001 and a batch size of 100. The number of epochs was set as 1000. The loss function for the training is Equation (8).

XGBoost is well suited for regression problems due to its inherent design and capabilities. Its core functionality revolves around building an ensemble of decision trees, each focusing on correcting the errors of its predecessors. This sequential learning approach allows XGBoost to effectively capture complex relationships within data and accurately predict continuous numerical values, making it a powerful tool for regression problems. The XGBoost model for ΔK has the same input and output as the DNN model. The XGBoost model utilised in this study adopted L1 and L2 regularisation terms, which were added to the loss function. The L1 and L2 regularisation terms are defined as follows:

(16) $$\begin{array}{cc} L1 & =\alpha \sum_{j=1}^{T}\left|w_{j}\right|, \end{array}$$

(17) $$\begin{array}{cc} L2 & =\frac{1}{2}\lambda \sum_{j=1}^{T}w_{j}^{2}, \end{array}$$

where *T* is the number of leaves in each tree, $w_{j}$ is the weight at the leaves, and α and λ are the controlling parameters for the L1 and L2 regularisation terms, respectively. The model’s parameters are as follows: the tree’s maximum depth is 8, the L1 regularisation term on weights is 0.5, the L2 regularisation term on weights is 0.3, the number of estimators is 3000, and the learning rate is 0.01. The hyperparameters were obtained using the package Hyperopt [30].

The XGBoost and DNN models were trained using the same straightening database, and the surrogate models from each machine learning model were generated. The database was partitioned for training, validation, and testing datasets. The ratios of the datasets were 64%, 16%, and 20% for training, validation, and testing, respectively. Table 1 shows the average loss of training, validation, and test datasets of the trained DNN and XGBoost models for each target geometry, where the loss is defined in Equation (8). The average losses show that the trained models can predict the change in the stiffness matrix without loss of generality.

## 4. Results and Discussion

The numerical experiments were conducted for the three target components in Figure 6 as mentioned in the previous section. For each target component, 1500 straightening strokes were simulated, and $K_{\mathrm{global}}$ and the machine learning models for ΔK were trained as shown in the left side of Figure 7. Ten randomly distorted geometries, which are not included in the training database, were generated for each component to evaluate the performance of the proposed straightening stroke decision algorithm.

The performances of the proposed algorithm with the machine learning model for ΔK and without it (which means only $K_{\mathrm{global}}$ was considered) were compared. The DNN model was selected for the performance comparison with the algorithm with $K_{\mathrm{global}}$ only.

**Figure 10:**
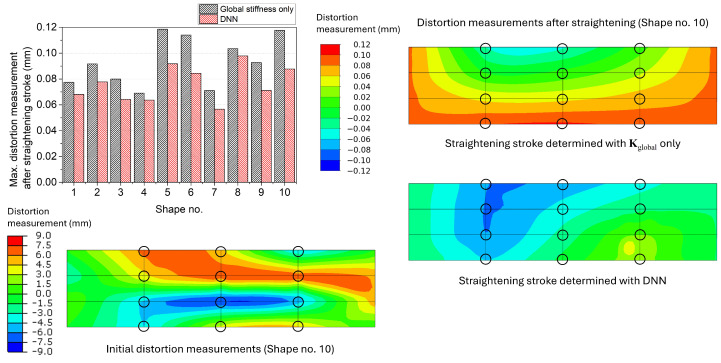
Maximum distortion measurements of the simple box after the straightening stroke decided using the global stiffness only and the surrogate model with the DNN model. The distortion measurement distributions of the initial and straightened geometries of Shape no. 10 are presented as an example. The black circles denote the locations of the straightening pins and distortion measurement.

The maximum distortion measurements of the simple box, after the straightening stroke decided using only $K_{\mathrm{global}}$ and the surrogate model with the DNN model, are depicted in Figure 10. For all ten randomly distorted shapes, the surrogate model with the DNN model showed better performance than the results that considered $K_{\mathrm{global}}$ only as the maximum distortion measurements of the straightened geometries were low with the surrogate model for all shapes. The average of the maximum distortion measurements after the straightening stroke was 0.0935 mm for the model with $K_{\mathrm{global}}$ only and 0.0751 mm for the surrogate model with the DNN model. The distortion measurement distributions of the simple box Shape no. 10 after the straightening strokes are presented on the right side of Figure 10. The distortion measurement distributions were considerably different since the model that considered $K_{\mathrm{global}}$ only could not take account of the change in the stiffness due to the geometry. In addition, this made the straightening stroke determined with only $K_{\mathrm{global}}$ less effective than the one determined by the surrogate model with the DNN model.

**Figure 11:**
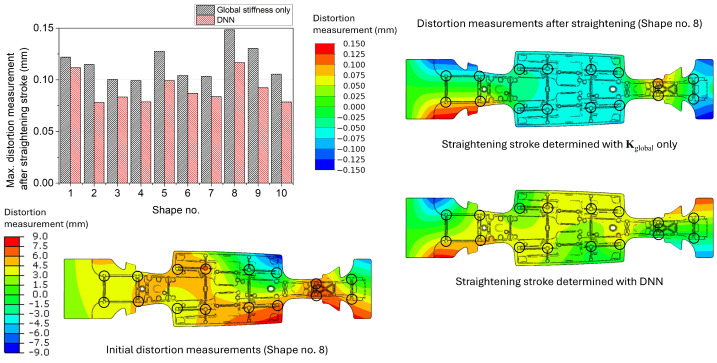
Maximum distortion measurements of the centre spine after the straightening stroke decided using the global stiffness only and the surrogate model with the DNN model. The distortion measurement distributions of the initial and straightened geometries of Shape no. 8 are presented as an example. The black circles denote the locations of the straightening pins and distortion measurement.

Figure 11 shows the maximum distortion measurements of the centre spine after the straightening stroke determined with only $K_{\mathrm{global}}$ and the surrogate model with the DNN model. The average of the maximum distortion measurements after the straightening stroke was 0.1156 mm for the model with $K_{\mathrm{global}}$ only and 0.0909 mm for the surrogate model with the DNN model. Similar to the simple box cases, the straightening strokes determined with the DNN model were more effective for all ten shapes. The magnitude of the distortion measurements for the straightened geometries was higher than the simple box since the centre spine has a complex geometry, and the number of straightening pins to determine is greater than for the simple box. On the right side of Figure 11, the distortion measurement distributions of the centre spine Shape no. 8 after the straightening are presented. Similar to the simple box, the overall distributions were different from each other, and the DNN model showed better performance than the model with only $K_{\mathrm{global}}$.

**Figure 12:**
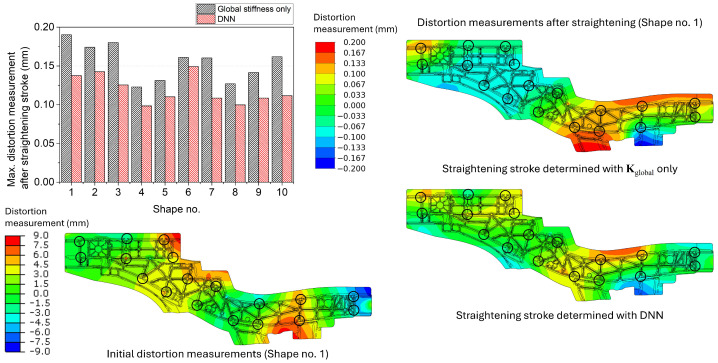
Maximum distortion measurements of the side member after the straightening stroke decided using the global stiffness only and the surrogate model with the DNN model. The distortion measurement distributions of the initial and straightened geometries of Shape no. 1 are presented as an example. The black circles denote the locations of the straightening pins and distortion measurement.

Similar results were derived for the side member as shown in Figure 12. The average of the maximum distortion measurements after the straightening stroke was 0.1550 mm for the model with $K_{\mathrm{global}}$ only and 0.1192 mm for the surrogate model with the DNN model. The side member showed the highest magnitude of the distortion measurements for the straightened geometries since the geometry’s size and complexity were the most significant among the target geometries. The distortion measurement distributions of the side member Shape no. 1 are presented on the right side of Figure 12. As shown in the simple box and the centre spine cases, the distortion measurement distributions were significantly different from each other since there is a difference in whether the current geometry was considered or not.

The straightening results derived from the model with only $K_{\mathrm{global}}$ and from the surrogate model with the DNN model showed that the surrogate model was superior to the model with only $K_{\mathrm{global}}$. Considering the current geometry’s effect on the component’s stiffness is crucial since the surrogate model provided more satisfactory straightening results from all target geometries. The surrogate model showed that the average of the maximum distortion measurements after the straightening stroke improved by 21.4% for all target geometries compared with the model with only $K_{\mathrm{global}}$. However, it should be noted that establishing the global stiffness matrix $K_{\mathrm{global}}$ is also an essential part of the surrogate model since the model is also based on global stiffness. An improper global stiffness matrix will make the algorithm decide the straightening stroke inappropriately, whether the effect of the current geometry is considered or not.

The straightening stroke determined from the naive L-BFGS-B mentioned in Section 3.2 could be treated as a local optimum solution since the naive L-BFGS-B does not utilise any surrogate model and attempts to find the optimal point from the finite element analysis results directly. In this study, the straightening results obtained from the naive L-BFGS-B were considered global optimum solutions because the search space was too broad to conduct a global optimisation process without a surrogate model. The performances of the surrogate models with the DNN model and with the XGBoost model were evaluated by comparing their results with those of the naive L-BFGS-B.

**Figure 13:**
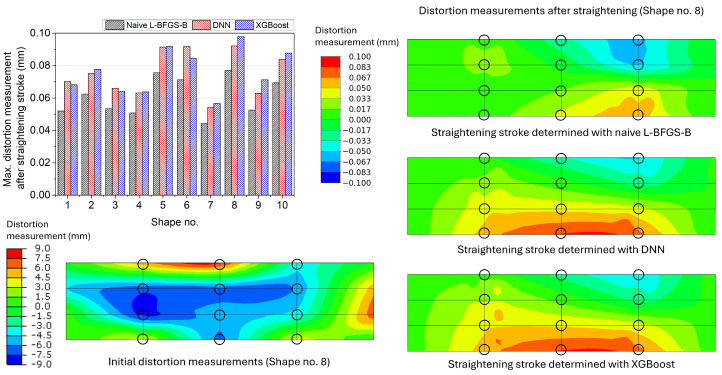
Maximum distortion measurements of the simple box after the straightening stroke decided using the naive L-BFGS-B and the surrogate models with the DNN model and with the XGBoost model. The distortion measurement distributions of the initial and straightened geometries of Shape no. 8 are presented as an example. The black circles denote the locations of the straightening pins and distortion measurement.

Figure 13 shows the maximum distortion measurements of the simple box after the straightening stroke determined using the naive L-BFGS-B and the surrogate models with the DNN model and with the XGBoost model. The average of the maximum distortion measurements after the straightening stroke was 0.0609 mm for the naive L-BFGS-B and 0.0763 mm for the surrogate model with the XGBoost model. The DNN model showed slightly better performance than the XGBoost model based on the straightening results of the simple box. The distortion measurement distributions of the simple box Shape no. 8 are presented on the right side of Figure 13. The surrogate models showed similar distortion measurement distributions, whilst the distribution of the naive L-BFGS-B was dissimilar from that of the surrogate models.

**Figure 14:**
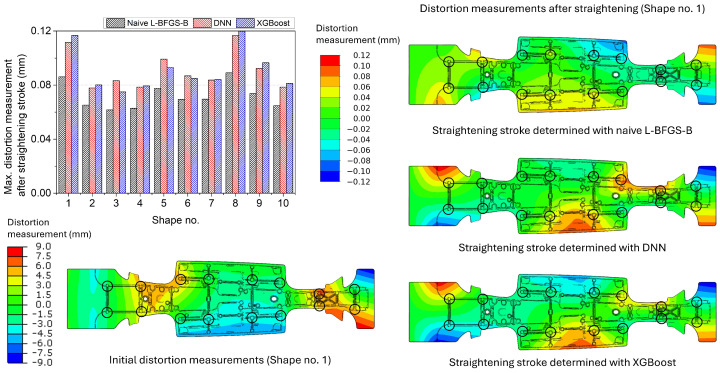
Maximum distortion measurements of the centre spine after the straightening stroke decided using the naive L-BFGS-B and the surrogate models with the DNN model and with the XGBoost model. The distortion measurement distributions of the initial and straightened geometries of Shape no. 1 are presented as an example. The black circles denote the locations of the straightening pins and distortion measurement.

The maximum distortion measurements of the centre spine, after the straightening stroke decided using the naive L-BFGS-B and the surrogate models with the DNN model and with the XGBoost model, are shown in Figure 14. The average of the maximum distortion measurements after the straightening stroke was 0.0720 mm for the naive L-BFGS-B and 0.0911 mm for the surrogate model with the XGBoost model. The DNN model performance was slightly better than that of the XGBoost model. However, the difference in the performance between the surrogate models was negligible. The distortion measurement distributions of the centre spine Shape no. 1 are shown on the right side of Figure 14. Similar to the simple box results, the distortion measurement distributions of the DNN and the XGBoost model were similar to each other. In contrast, the naive L-BFGS-B showed a different pattern of distribution.

**Figure 15:**
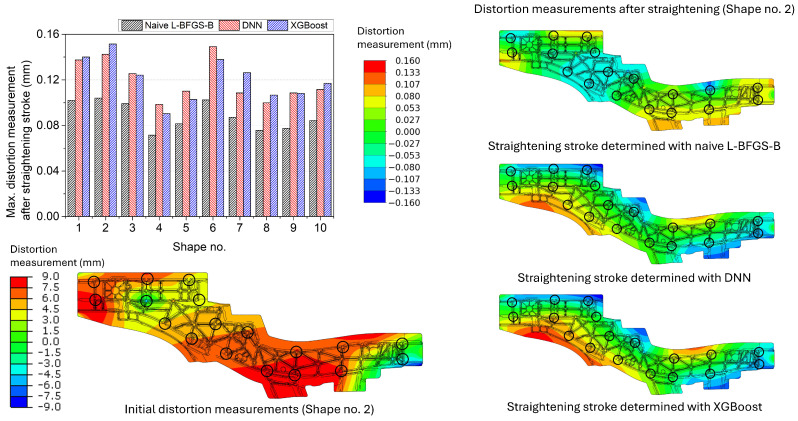
Maximum distortion measurements of the side member after the straightening stroke decided using the naive L-BFGS-B and the surrogate models with the DNN model and with the XGBoost model. The distortion measurement distributions of the initial and straightened geometries of Shape no. 2 are presented as an example. The black circles denote the locations of the straightening pins and distortion measurement.

The side member’s maximum distortion measurements after the straightening stroke decided using the naive L-BFGS-B and the surrogate models with the DNN model and with the XGBoost model are shown in Figure 15. The average of the maximum distortion measurements after the straightening stroke was 0.0886 mm for the naive L-BFGS-B and 0.1205 mm for the surrogate model with the XGBoost model. The magnitude of the distortion measurements was the largest among the target geometries for the same reason discussed above. Like the centre spine, the DNN model showed slightly better performance than the XGBoost model. The distortion measurement distributions of the side member Shape no. 2 are depicted on the right side of Figure 15. The straightening behaviour was similar between the DNN and XGBoost models, whereas the naive L-BFGS-B showed different behaviour.

The maximum distortion measurements after the straightening stroke for each method and model are organised in Appendix A. Student’s *t*-tests were conducted to evaluate the efficiency of each proposed model for the straightening process. The naive L-BFGS-B was excluded from the tests since it was used to make reference results for each target geometry because the naive L-BFGS-B can be considered to determine the optimum straightening stroke. In the statistical analysis, the significance level was set to be α=0.05 and the standard deviations were assumed to be different.

Table 2 shows the *p*-values of the two-tailed tests with the null hypothesis of the same mean for the surrogate models with the DNN and XGBoost. In Table 2, $H_{0}$ is the null hypothesis, and $\mu_{1}$ and $\mu_{2}$ are the means of the maximum distortion measurements after the straightening stroke for the surrogate models with the DNN and XGBoost, respectively. For all geometries, the *p*-values are greater than the significance level α, which means that the null hypothesis should not be rejected and the means of the maximum distortion measurements were statistically identical.

Table 3 shows the *p*-values of the left-tailed tests with the null hypothesis of $H_{0}^{a}$: $\mu_{1}\geq \mu_{2}$, where $\mu_{1}$ and $mu_{2}$ are the means of the maximum distortion measurements after the strengthening stroke for the surrogate model with the DNN and the model with the global stiffness only, respectively. The *p*-values are less than the significance level for all geometries, which means the null hypothesis should be rejected. It shows that the straightening performances were statistically better with the DNN model since the mean is smaller for the surrogate model with the DNN model.

The surrogate model with global stiffness could only be used for straightening. However, the straightening performance can be improved when the DNN and XGBoost models are utilised in the surrogate model with the global stiffness matrix, and the statistical analysis results support this. In addition, the maximum distortion measurements after the straightening stroke were reduced by 26.6% on average when the DNN or XGBoost model was adopted in the surrogate model.

Compared to the straightening results from naive L-BFGS-B, the maximum distortion measurements after the straightening stroke from DNN and XGBoost models differed by 28.0% and 29.3%, respectively, on average. The surrogate models with the DNN model and with the XGBoost model showed similar performances for the target geometries, while the DNN model showed slightly better performance on average. On the other hand, it cannot be said that the DNN model is always preferable to the XGBoost model since the DNN model did not show better straightening performance for all cases. In addition, the statistical analysis results indicate that the performances of the DNN and XGBoost models were statistically identical. The straightening results showed that the DNN and XGBoost models are adequate to model ΔK, the change in the component’s stiffness. In addition, the surrogate models could decide the straightening strokes so that the maximum distortion after the straightening becomes 0.02% of the largest dimension of each target geometry.

The distortion measurement distributions from the surrogate models were similar for all target components, while the distribution from the naive L-BFGS-B was different. This shows that the DNN and XGBoost models estimated ΔK similarly, whilst the naive L-BFGS-B estimated it directly from the finite element analyses. The estimated stiffness matrix can differ from the real stiffness matrix of the current geometry. However, the naive L-BFGS-B is computationally expensive, which means that it cannot be utilised to operate the straightening process. On the contrary, the surrogate model can provide admissible solutions for the straightening process with an adequately fitted $K_{\mathrm{global}}$ and a trained machine learning model.

## 5. Conclusions

This paper proposes a method for straightening distorted giga-cast thin-walled components. The straightening process, which corrects the distorted part by pressing it with numerous straightening pins, is described, and the concept of a straightening machine to carry out the process is suggested. An algorithm which decides the straightening stroke is suggested. The straightening stroke decision algorithm has two primary models: the polynomial model representing the component’s global stiffness and the machine learning model for the change in the stiffness due to the distorted geometry. Two machine learning models, DNN and XGBoost, were selected to model ΔK. The numerical experiments, which make distortion and straighten the component with the finite element analyses, were conducted to evaluate the performance of the proposed algorithm. Three components were adopted for the numerical experiments: the simple box, centre spine, and side member. From the results of the numerical experiment, the conclusions can be summarised as follows:

- The effect of the current geometry should be considered when deciding the straightening stroke to make the straightening strokes effective. The global stiffness matrix can be used alone for straightening, but the determined straightening strokes might be less effective.
- The proposed DNN and XGBoost models adequately modelled the stiffness change due to the current geometry. Their performances on the straightening stroke decision were similar; meanwhile, the DNN model showed slightly better performance.
- Regarding the straightening results from the naive L-BFGS-B as the optimum, the maximum distortion measurements after the straightening strokes decided from the surrogate models differed by 28.7% on average. The surrogate models could provide admissible straightening stroke results, considering that the naive L-BFGS-B process is computationally expensive, which means that it is not appropriate to operate the straightening process.
- The surrogate models decided the straightening strokes so that the maximum remaining distortion became 0.02% of the largest dimension of each target geometry for all target components. The suggested straightening method and algorithm were suitable for straightening distorted large thin-walled components.

However, the proposed straightening method was only examined using numerical experiments. As future work, we suggest developing a real straightening machine and applying the straightening process to real large-sized thin-walled die-cast components to examine the proposed method for actual application. In addition, we only considered cases in which straightening pin locations and distortion measurement locations were identical. In future work, cases with different straightening pin locations and distortion measurements need to be considered and analysed.

## Figures and Tables

**Table 1:** Average loss of training, validation, and test datasets of the trained DNN and XGBoost models for each target geometry.

Unit: $10^{-3}$ mm						
	**Simple Box**		**Centre Spine**		**Side Member**	
**Dataset**	**DNN**	**XGBoost**	**DNN**	**XGBoost**	**DNN**	**XGBoost**
Training	0.4715	0.4648	7.0851	7.1964	8.4046	8.4928
Validation	0.4918	0.4937	7.0366	7.2356	8.5231	8.7319
Test	0.4779	0.4783	7.2750	7.2438	8.5846	8.5119

**Table 2:** *p*-values of the two-tailed tests with the null hypothesis of the same mean for the surrogate models with the DNN and XGBoost.

$H_{0}^{a}$: $\mu_{1}=\mu_{2}$			
	**Simple Box**	**Centre Spine**	**Side Member**
*p*-value	0.8372	0.9718	0.8752

**Table 3:** *p*-values of the left-tailed tests with the null hypothesis of $H_{0}^{b}$: $\mu_{1}\geq \mu_{2}$, where $\mu_{1}$ and $mu_{2}$ are the means of the maximum distortion measurements after the strengthening stroke for the surrogate model with the DNN and the model with the global stiffness only, respectively.

$H_{0}^{b}$: $\mu_{1}\geq \mu_{2}$			
	**Simple Box**	**Centre Spine**	**Side Member**
*p*-value	0.0123	0.0009	0.0007

## Data Availability

Data are contained within the article.

## Appendix A. Tables for Maximum Distortion Measurements after the Straightening Stroke

The following are the tables of maximum distortion measurements after the straightening stroke decided using the naive L-BFGS-B, the global stiffness only, and the surrogate models with the DNN and XGBoost models.

**Table A1:** Maximum distortion measurements after the straightening stroke for the simple box.

Unit: mm				
**Shape No.**	**Naive L-BFGS-B**	**$K_{\mathrm{global}}$ Only**	**DNN**	**XGBoost**
1	0.0520	0.0774	0.0702	0.0681
2	0.0625	0.0917	0.0752	0.0778
3	0.0534	0.0800	0.0660	0.0642
4	0.0507	0.0690	0.0631	0.0637
5	0.0756	0.1185	0.0914	0.0918
6	0.0712	0.1140	0.0918	0.0844
7	0.0442	0.0710	0.0541	0.0567
8	0.0770	0.1035	0.0921	0.0979
9	0.0526	0.0927	0.0628	0.0712
10	0.0693	0.1177	0.0837	0.0877

**Table A2:** Maximum distortion measurements after the straightening stroke for the centre spine.

Unit: mm				
**Shape No.**	**Naive L-BFGS-B**	**$K_{\mathrm{global}}$ Only**	**DNN**	**XGBoost**
1	0.0862	0.1220	0.1116	0.1168
2	0.0652	0.1149	0.0780	0.0802
3	0.0617	0.1003	0.0833	0.0750
4	0.0628	0.0993	0.0785	0.0794
5	0.0776	0.1276	0.0993	0.0930
6	0.0694	0.1042	0.0869	0.0850
7	0.0696	0.1032	0.0837	0.0840
8	0.0891	0.1483	0.1166	0.1199
9	0.0738	0.1303	0.0923	0.0966
10	0.0647	0.1055	0.0785	0.0813

**Table A3:** Maximum distortion measurements after the straightening stroke for the side member.

Unit: mm				
**Shape No.**	**Naive L-BFGS-B**	**$K_{\mathrm{global}}$ Only**	**DNN**	**XGBoost**
1	0.1021	0.1902	0.1375	0.1401
2	0.1040	0.1742	0.1425	0.1514
3	0.0992	0.1801	0.1255	0.1242
4	0.0715	0.1230	0.0984	0.0905
5	0.0815	0.1313	0.1102	0.1028
6	0.1024	0.1609	0.1493	0.1380
7	0.0872	0.1603	0.1086	0.1263
8	0.0758	0.1268	0.0998	0.1067
9	0.0776	0.1415	0.1085	0.1082
10	0.0844	0.1619	0.1117	0.1170
